# Global DNA methylation of peripheral blood leukocytes from dogs bearing multicentric non-Hodgkin lymphomas and healthy dogs: A comparative study

**DOI:** 10.1371/journal.pone.0211898

**Published:** 2019-03-25

**Authors:** Tatiane Moreno Ferrarias Epiphanio, Natália Coelho Couto de Azevedo Fernandes, Tiago Franco de Oliveira, Priscila Assis Lopes, Rodrigo Albergaria Réssio, Simone Gonçalves, Náyra Villar Scattone, Marcello Vannucci Tedardi, Leslie Domenici Kulikowski, Jullian Damasceno, Ana Paula de Melo Loureiro, Maria Lucia Zaidan Dagli

**Affiliations:** 1 Laboratory of Experimental and Comparative Oncology, Department of Pathology, University of São Paulo, São Paulo, São Paulo, Brazil; 2 Department of Pathology, Adolfo Lutz Institute, São Paulo, São Paulo, Brazil; 3 Department of Pharmacoscience, Federal University of Health Sciences of Porto Alegre, Porto Alegre, Rio Grande do Sul, Brazil; 4 Veterinary Laboratory, Veterinary Image Institute, IVI, São Paulo, São Paulo, Brazil; 5 Veterinary Hemotherapy Center, Hemovet, São Paulo, São Paulo, Brazil; 6 Cytogenomic Laboratory, Department of Pathology, University of São Paulo, São Paulo, São Paulo, Brazil; 7 Department of Clinical and Toxicological Analysis, University of São Paulo, São Paulo, São Paulo, Brazil; Chuo University, JAPAN

## Abstract

Non-Hodgkin lymphomas are among the most common types of tumors in dogs, and they are currently accepted as comparative models of the disease in humans. Aberrant patterns of DNA methylation seem to play a key role in the development of hematopoietic neoplasms in humans, constitute a special mechanism of transcriptional control, and may be influenced by genetic and environmental factors. Blood leukocyte DNA global methylation has been poorly investigated in dogs. The aim of this study is to examine whether peripheral blood global DNA methylation is associated with canine multicentric lymphomas. Peripheral venous blood samples from ten healthy dogs and nine dogs bearing multicentric lymphomas were collected, and the buffy coat was separated. Global DNA methylation was analyzed by High Performance Liquid Chromatography (HPLC) and immunocytochemistry (ICC). In both analyses, leukocytes from dogs with lymphoma presented lower global DNA methylation than in healthy dogs (HPLC: *p* = 0.027/ 5MeCyt immunoreactivity scores: *p* = 0.015). Moderate correlation was observed between the results obtained by HPLC and ICC (correlation coefficient = 0.50). For the identification of differently methylated genes between both groups, the Infinium Human Methylation (HM) EPIC BeadChip (850K) was used. Of the 853,307 CpGs investigated in the microarray, there were 34,574 probes hybridized in the canine samples. From this total, significant difference was observed in the methylation level of 8433 regions, and through the homologous and orthologous similarities 525 differently methylated genes were identified between the two groups. This study is pioneer in suggesting that dogs bearing non-Hodgkin lymphoma presented DNA global hypomethylation of circulating leukocytes compared with healthy dogs. Although canine samples were used in an assay developed specifically for human DNA, it was possible to identify differently methylated genes and our results reiterate the importance of the use of peripheral blood leukocytes in cancer research and possible new biomarkers targets.

## Introduction

Neoplastic processes are the leading cause of death in adult dogs in North America [1]. Lymphomas are among the most common types of tumors in dogs, and they are responsible for 83% of all canine hematopoietic malignancies [2,3]. The annual rate is 22.9 per 100,000 live births in females and 19.1 per 100,000 live births in males for canine lymphoma [4]. The disease shares many features with human lymphoma, including clinical presentation, biological behavior, tumor genetics, and treatment response [5]. Dogs with high-grade multicentric lymphoma generally show painless peripheral lymphadenopathy and infrequently present clinical signs associated with the effects of tumor infiltration [3].

Etiology of canine lymphomas is likely multifactorial. Chromosomal aberrations, germline and somatic genetic mutations, altered oncogene/tumor suppressor gene expression, and epigenetic changes have been reported in dogs [6–9]. Heritable risk factors causing the disease were introduced because certain dog breeds presented a prevalence of immunophenotypic subtypes of lymphoma [10,11]. Several environmental factors have been associated with lymphomas. Exposure to herbicides, waste incinerators, polluted sites, and radioactive waste can be considered risk factors for canine lymphoma [12–14]. Dogs with spontaneously arising lymphoma represent a large animal model of naturally occurring lymphoma in a species that shares the human household environment and potential carcinogen exposure [12].

Epigenetic markers are influenced by a mix of genetic and environmental variation [15]. There are several mechanisms involving genomic instability and epigenomic aberrations, with loss or gain of gene function that interfere with tumor suppression/prevention or oncogenesis [16]. In cancer, growing evidence indicates an epigenome-wide disruption that involves hypomethylation of large regions of the genome, which induces genomic instability indicated by increased chromosomal rearrangements, mitotic recombination, and aneuploidy [17]. In contrast, DNA hypermethylation predominantly involves CpG islands (promoter regions) and has been shown to result in abnormal silencing of several tumor suppressor genes in most types of cancer [18–20]. Both mechanisms are favorable to carcinogenesis and tumor progression.

In humans, DNA hypomethylation seems to be an important factor in the pathogenesis of mature B-cell neoplasms (MBCN), described in tumor tissue and peripheral blood compared with both normal tissue and blood [21,22]. Although any tissue can be used to detect novel regions of differential methylation associated with a cancer phenotype, harvesting tumor tissue is invasive and cannot be routinely performed. Therefore, peripheral blood leukocyte DNA was evaluated as a biomarker for prevention, early detection, and cancer monitoring, and offers the advantage of being a readily available tissue [23–26]. Several studies have reported aberrant global methylation profiles in human peripheral blood with different cancers: MBCN, leukemia, colorectal cancer, breast cancer, hepatic cancers, and urothelial cancers [16, 22, 25, 27–30].

In canine species, researches on global methylation effects are currently increasing, especially in cancer. In a recent investigation, Ferraresso and colleagues suggested an important role of DNA methylation in canine diffuse large B-cell lymphomas (DLBCL), where aberrancies in transcription factors were frequently observed, suggesting an involvement during tumorogenesis and they hypothesized that the accumulation of aberrant epigenetic changes resulted in a more aggressive behavior of the tumor [31]. Another study proved that global DNA hypomethylation was predominant in canine cutaneous high-grade mast cell tumors by immunohistochemical detection, and genomic hypomethylation was a feature of neoplastic cells obtained from dogs with malignant lymphoproliferative disorders [32,9]. Few studies have investigated perturbations of DNA methylation at single gene level, such as *p16*, *DLC1*, *ABCB1*, and *FHIT* [33–36]. Epigenetic deregulation of *TFPI-2*, leading to its reduced expression in canine diffuse large B-cell was demonstrated [37]. However, there are no published examinations of possible global measures of peripheral blood-derived DNA methylation in canine lymphoma. The aims of this study were to investigate whether a global measure of DNA methylation dispersed over a large portion of the genome could be detected in canine peripheral blood DNA, and whether such a measure was associated with development of lymphoma. Moreover, genome-wide characterization of lymphoma epigenome was performed with a human CpG microarray platform targeting more than 850,000 CpG regions. This study contributes to detection of epigenetic alterations in peripheral blood, and offers new possibilities of potential biomarkers for canine non-Hodgkin lymphoma.

## Materials and methods

### Patient details and ethics

Ethical approval for this study was granted by the Ethics Committee on the Use of Animals of the School of Veterinary Medicine (process number: 1747271015) and School of Medicine of the University of São Paulo (process number: 024/16). Participants were selected and separated into two groups (control and lymphoma), with 10 and nine dogs in each group respectively (19 dogs in total). The Control group was enrolled in the dog kennel from Veterinary Hemotherapy Center “Hemovet”, minimum age of 5 years, both sexes, and defined breed. Anamnesis, physical examination, complete blood count, and samples from canine lymph node by fine-needle aspiration biopsy (FNAB) confirmed to be healthy. The Lymphoma group was enrolled in the Veterinary Image Institute “IVI”, minimum age of 5 years, composed of both sexes, with or without defined breed, bearing previously untreated multicentric lymphoma diagnosed by FNAB through analysis of panoptic-stained smears. Cases with more than 50% of blastic population, or more than 80% of monomorphic population, were considered neoplastic and classified as low-grade or high-grade lymphoma according to updated Kiel classification [38]. The exclusion criteria adopted were as follows: morphologically abnormal circulating leukocytes, history of neoplasms and/or other neoplasm, use of medications (corticosteroids and/or chemotherapy), and other concomitant diseases.

### STAGE 1: Genome-wide methylation with HPLC and ICC

#### Sampling

Peripheral venous blood samples (8 mL) from 19 dogs were collected in tubes with EDTA and centrifuged at 3000g for 10 min at 4°C. The leukocyte fraction was then collected, and each sample was separated into two similar aliquots. The first aliquot was added to a vial with preservative fluid (Becton Dickinson, TriPath Imaging, Burlington, NC, USA) for preparation of cell block (CB). Samples were refrigerated until analysis for a maximum of 15 days. Another aliquot was stored at -80°C for subsequent DNA extraction. In addition to cytological smear, another sample from the same lymph node of dogs bearing lymphoma was collected by FNAB and flushed into a vial with preservative fluid (Becton Dickinson, TriPath Imaging, Burlington, NC, USA). The aspirations proceeded until the preservative liquid acquired turbidity and the samples were then refrigerated until analysis for a maximum of 15 days.

#### CB and immunophenotyping

Samples stored in the preservative fluid were centrifuged for 10 min in 1218g. The rejected supernatant and the resulting pellet were fixed with 200μL of Bouin’s solution (2,4,6-Trinitrophenol 1,3%, formalin 40%, glacial acetic acid 100%) and centrifuged (1754g, 15 min) (fixed sediment method–FSM). The pellets were placed in a cassette and stored in 10% formalin-buffered solution and then embedded in paraffin wax and trimmed in 3μm-thick sections for H&E staining and immunocytochemistry [39]. Antigen retrieval was conducted with citrate buffer 10mMpH6.0 in a pressure cooker for 3 min at 120°C. Endogenous peroxidase was blocked with 6% hydrogen peroxide for a minimum of 30 min. Immunophenotyping was established from CBs made from lymph nodes of the neoplastic group. Primary antibodies anti-CD3 (polyclonal, 1:400) (Dako, Carpinteria, CA, USA), anti-PAX5 (BC-24, 1:200) (Biocare Medical, Concord, CA, USA), anti-CD79a (HM57, 1:100) (Dako, Carpinteria, CA, USA), and anti-Ki67 (MIB-1, 1:100) (Dako, Carpinteria, CA, USA) were diluted in bovine serum albumin 1% Na3N 0.1% on PBS (pH 7.4), followed by overnight incubation (18 h) at 4°C. Signal amplification with Picture Max Kit (Life Technologies, Carlsbad, CA, USA) secondary peroxidase short polymer system was conducted for 30 min at 37°C. After development with 100mg of 3,3’-diaminobenzidine (D-5637; Sigma, St. Louis, MO, USA), the samples were diluted on PBS (pH 7.4) for 5 min at 37°C. The samples were then counterstained with Harris Hematoxylin for 30s at room temperature, followed by dehydration and slide mounting with synthetic resin.

#### HPLC (high-performance liquid chromatography)

The procedure used for DNA extraction and purification was performed following the protocol from Gentra Puregene DNA extraction kit (QIAGEN Sciences, Maryland, USA). The previously collected leukocyte fractions were added to 6mL of erythrocyte lysis solution (*RBC Lysis Solution*, Gentra Puregene kit) following 10 min of incubation at room temperature; samples were then centrifuged at 2000g for 5 min at 4°C, and supernatant carefully discarded. The samples were added to 3 mL of cell lysis solution (*Cell Lysis Solution*, Gentra Puregene kit) and 50 μL of ribonuclease A (15 mg/mL). They were subsequently incubated at 37°C for 60 min, protein was precipitated by addition of 1 mL of protein precipitation solution (*Protein Precipitation Solution*, Gentra Puregene kit), and centrifuged at 3000g for 10 min at 4°C. The supernatant was poured into a tube containing 5 mL of cold 2-isopropanol, and the precipitated DNA was collected by centrifugation in 2000g for 5 min at 4°C. The DNA was then washed with 3 mL of 70% ethanol, dried, and resuspended in 200 μL of 0.1 mM deferoxamine solution. DNA concentration was determined by measuring UV absorption at λmax: 260nm (Libra S12 Spectrophotometer, Biochrom, Cambridge, UK), and DNA purity was assessed by the UV absorbance ratio at λmax: 260/λmax: 280nm. Aliquots of 10 μg DNA samples in 0.1 mM deferoxamine solution were added to 2.5 μL of Tris-HCl/MgCl_2_ buffer (200 mM; pH 7.4) and 1 unit of DNase I. The samples were incubated at 37°C for 60 min. Phosphodiesterase I (0.001 units) and alkaline phosphatase (1.2 unit) were then added, and incubation continued for another 60 min at 37°C. At the end of the second incubation, the final volume (20 μL) was centrifuged at 9300g for 10 min. Aliquots of 10 μL were injected in the HPLC-UV analytical system (*Shimadzu Corporation*, Kyoto, Japan) for quantification of global DNA methylation, expressed as percent 5-methyl-2’-deoxycytidine (5MeCyt), determined using the following equation:

$$5‐methyl‐deoxycytidine(\%)=\frac{5‐methyl‐{deoxycytidine}_{\left(nmol\right)}\times 100}{5‐methyl‐{deoxycytidine}_{\left(nmol\right)}+{deoxycytidine}_{\left(nmol\right)}}$$

#### Immunocytochemistry

CBs produced by leukocytes from control and neoplastic dogs were submitted to the same protocol previously described for immunophenotyping, but the anti-5-methylcytosine (5MeCyt) (33D3, 1:100) (Abcam, Cambridge, MA, USA) was used as primary antibody. The global DNA methylation content was evaluated using Image-Pro Plus Analysis System. Digital photomicrographs were taken under the same light conditions. Ten distinct microscopic fields (40× objective) of each sample were analyzed. The nuclei of all leukocytes of each field were evaluated according to the immunostaining intensity. The grades of staining intensity were four-tiered as follows: 0: negative staining, 1: weak staining, 2: medium staining, and 3: strong staining. The H-score represented the sum of the mean value in each grade multiplied by the proportion of positive cells for each sample [40]. For each sample of leukocytes, the percentages of the immunostaining score patterns were considered in the statistical analysis.

#### Statistical analysis

R 3.3.2 software was used for the statistical analysis. After the normality test, by Kolmogorov-Smirnov test, the peripheral blood global DNA methylation quantities obtained by the HPLC and ICC methods for the control and lymphoma groups were compared using the Student’s t- test. The Pearson’s correlation test was used to compare the results from the HPLC and ICC methods. Two-way ANOVA was performed to evaluate combined influence of sex or age and lymphoma/control group over global methylation and immunoreactivity expression (to perform the test, animals were grouped in animals until 10 years and elder dogs). Mann-Whitney test compared lymphocytes count between healthy and lymphoma groups and Spearman correlation was used to compare lymphocytes count and global DNA methylation quantities obtained by the HPLC and ICC methods.

### STAGE 2: Infinium HM850 BeadChip DNA methylationEPIC analysis

#### Sampling

DNA extracted from buffy coats of four healthy dogs and four dogs bearing high grade B-cell lymphoma were selected according to age as control and experimental groups, respectively.

#### Illumina 850 K methylation

Based on the similarities between human and dog and on the concepts of homology and orthology, Infinium HM850 BeadChip DNA methylationEPIC analysis (Illumina, California, USA) was chosen, representing the methylation state of over 850 K CpG sites [41]. This platform interrogates 853,307 positions of methylation by sample, located in enhancer regions, body gene, and intergenic regions [42]. DNA aliquots extracted in the HPLC procedure were used. The quality and quantity of DNA were evaluated by the Quant-iT Picogreen ds DNA test and measured on Qubit fluorometer (Life Technologies, NY, USA). 200 ng of DNA was bisulfite converted with the EZ-96 DNA Methylation-Gold Kit, used according to the manufacturer’s protocol (Zymo Research, Orange, CA, USA). Briefly, 3**μ**l of bisulfite converted DNA were denatured, neutralized, and amplified; after precipitation with isopropanol, the DNA was collected by centrifugation and resuspended in buffer for hybridization to HM 850 BeadChip at 48°C for 16 h, followed by single nucleotide extension according to the Illumina Infinium HD Methylation protocol. Next, the incorporated nucleotides were labelled with biotin (ddCTP and ddGTP) and 2,4-dinitrophenol (DNP) (ddATP and ddTTP), and the BeadChip was then scanned using a Illumina HiScan scanner (Illumina, San Diego, CA, USA).

#### Statistical analyses

The intensities for each probe were extracted using the GenomeStudio (Illumina). The data obtained from reading the red and green channels were converted into a methylated and unmethylated signal. After generation of this signal, a value β between 0 and 1 was provided (value 1 means totally methylated) [43,44]. Next, the first step included filtering the probe with low fluorescence detection; probes that showed *p*-values> 0.05 and beads <3 counts in 5% of the samples. Quality control check was performed by means of the distribution of the signals obtained for methylated and non-methylated probes and the use of multivariate analysis—multidimensional scaling (MDS).

Beta methylation values were converted logarithmically into M-values. Statistical analysis was performed using the M-values because they were more homogeneous and less dispersed in the data [44]. The Bayes test (Empirical Bayes Statistics for Differential Expression) from limma package of the Bioconductor [45] was used for this analysis. The differential profile of methylation between the sample groups and the probes was obtained after correlation of *p*-value (*p*<0.05). MVP (Methylation Variable Positions) is a method that seeks differential methylation at a single CpG site, and its analysis consists in calculating the differential *p*-value of methylation. This analysis also shows how many MVPs are found between the groups studied, and the data are filtered by a *p*-value of interest, including for each probe. Probe target sequences interrogated by the HM850 array were extracted from the annotation library IlluminaHuman- Methylation850k in Bioconductor.

In the comparative analyses, the samples were divided into control and lymphoma groups. The data previously generated were used to study the differences between these two groups. In order to examine the inference of homologous and orthologous genes between the human and canine species, the UCSC Genome Browser [46] and Ensembl [47] bioinformatics tools were used, respectively. The dog (*canFam3*.*1*) reference genome was used for inferences. Functional characterization of genes differentially methylated was performed by enrichment approach topGO, using R software and Gene Set Analysis for DNA Methylation Datasets (methylGSA): via IlluminaHumanMethylationEPICanno.ilm10b2.hg19.

## Results

### STAGE 1: Genome-wide methylation with HPLC and ICC

#### Sampling

Ten dogs with mean age of 7.7 years (5–11 years), 3 males and 7 females, belonged to the Control group. Dog breeds were Golden Retriever (90%) and Boxer (10%). Nine multicentric lymphoma cases were identified, median age 10,5 years (5–13 years), 3 males and 6 females. Dog breeds were Golden Retriever (1), Labrador (1), American Pittbull (1), Dachshund (1), Swiss Sheppard (1), Maltese (1), and mongrel (3), represented in Table 1. Complete clinical and pathological features of dogs are reported in S1 Table [48].

**Table 1:** Demographic characteristics, lymphocytes count circulating of participants and tumors classification (*n* = 19).

	Lymphoma Group (*n* = 9)	Control Group (*n* = 10)
**Sex**		
Male	3	3
Female	6	7
**Breed**		
Golden Retrievers	1	9
All other breeds	8	1
**Age (years)**		
Average ± SD	10,5 ± 3,04	7,7 ± 2,62
Lymphocytes Count (/μL)*		
Average ± SD	4.827 ± 8.419	2.141 ± 660
**Classification**		
*High-grade B-cell Lymphoma*	**6**	
Subgroups		
Centroblastic monomorphic	3	
Plasmocytoid	1	
Immunoblastic	1	
Centroblastic polymorphic small and large cell	1	
*Low-grade B-cell Lymphoma*	**1**	
Subgroup		
Centroblastic-centrocytic	1	
*High-grade T-cell Lymphoma (subtypes)*	**2**	
Subgroups		
Lymphoblastic	1	
Plasmocytoid	1	

*Reference interval [48]: 1.000–4.000/ μL

#### CB and immunophenotyping

Seven cases were diagnosed as B-cell lymphomas (6 high-grade B-cell and 1 low-grade B-cell lymphomas) and two were classified as high-grade T-cell lymphomas based on morphological features (lymphocyte size, nuclear and cytoplasm features, presence of atypia and mitotic figures), with immunophenotyping and proliferation index through Ki-67. All lymphoma patients presented with peripheral lymphadenopathy. No dog presented atypical lymphocytes in blood smear examination. B-cell lymphomas were classified into five major subtypes: centroblastic monomorphic (n = 3), plasmocytoid (n = 1), immunoblastic (n = 1), centroblastic polymorphic small and large cell (n = 1), and centroblastic-centrocytic (n = 1). T-cell lymphomas were classified as lymphoblastic (n = 1) and plasmocytoid (n = 1) (Table 1).

#### HPLC

**Fig 1:**
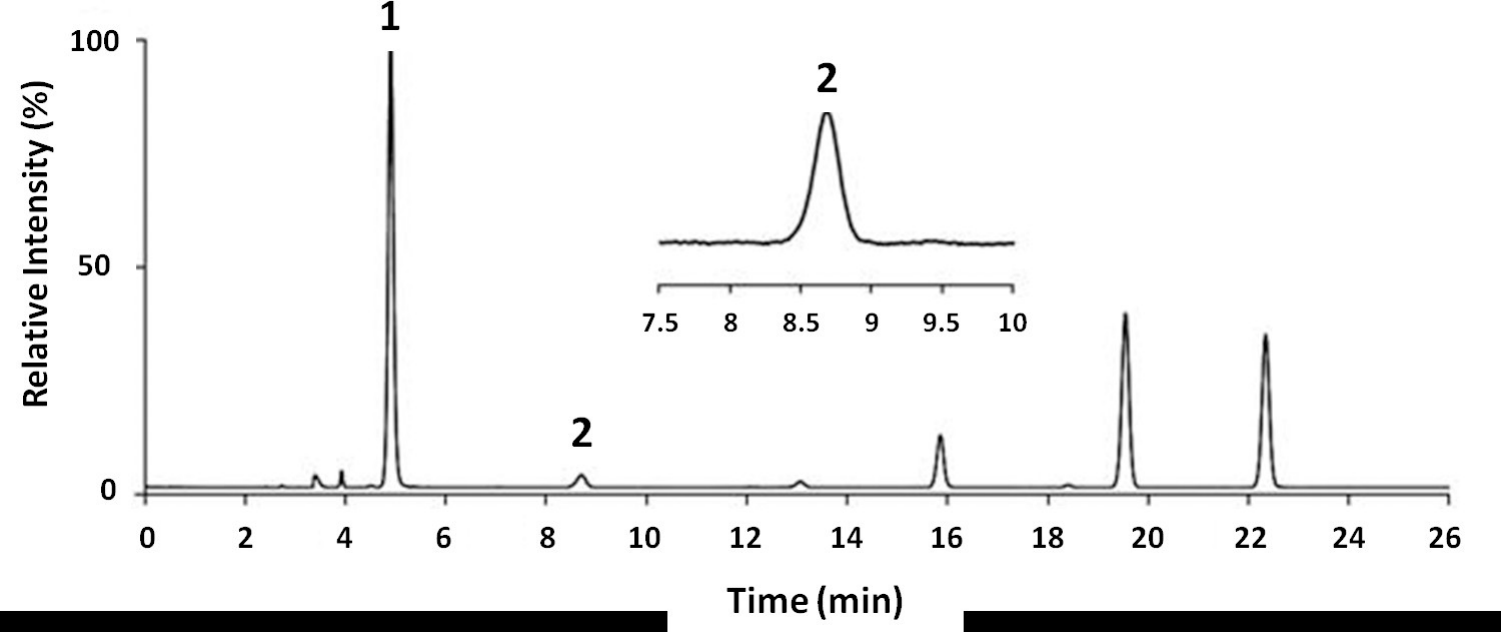
Chromatogram of a dog bearing lymphoma by HPLC-DAD (λ = 260 and 286 nm). 1: quantity of dC. 2: quantity of 5MeCyt. The curve area yields the numerical values to be entered into the formula for calculating the percentage of overall DNA methylation (5MeCyt%).

Quantification of global DNA methylation was measured according to the area obtained in the chromatograms referring to deoxycytidine and 5MeCyt, as shown in Fig 1.

**Fig 2:**
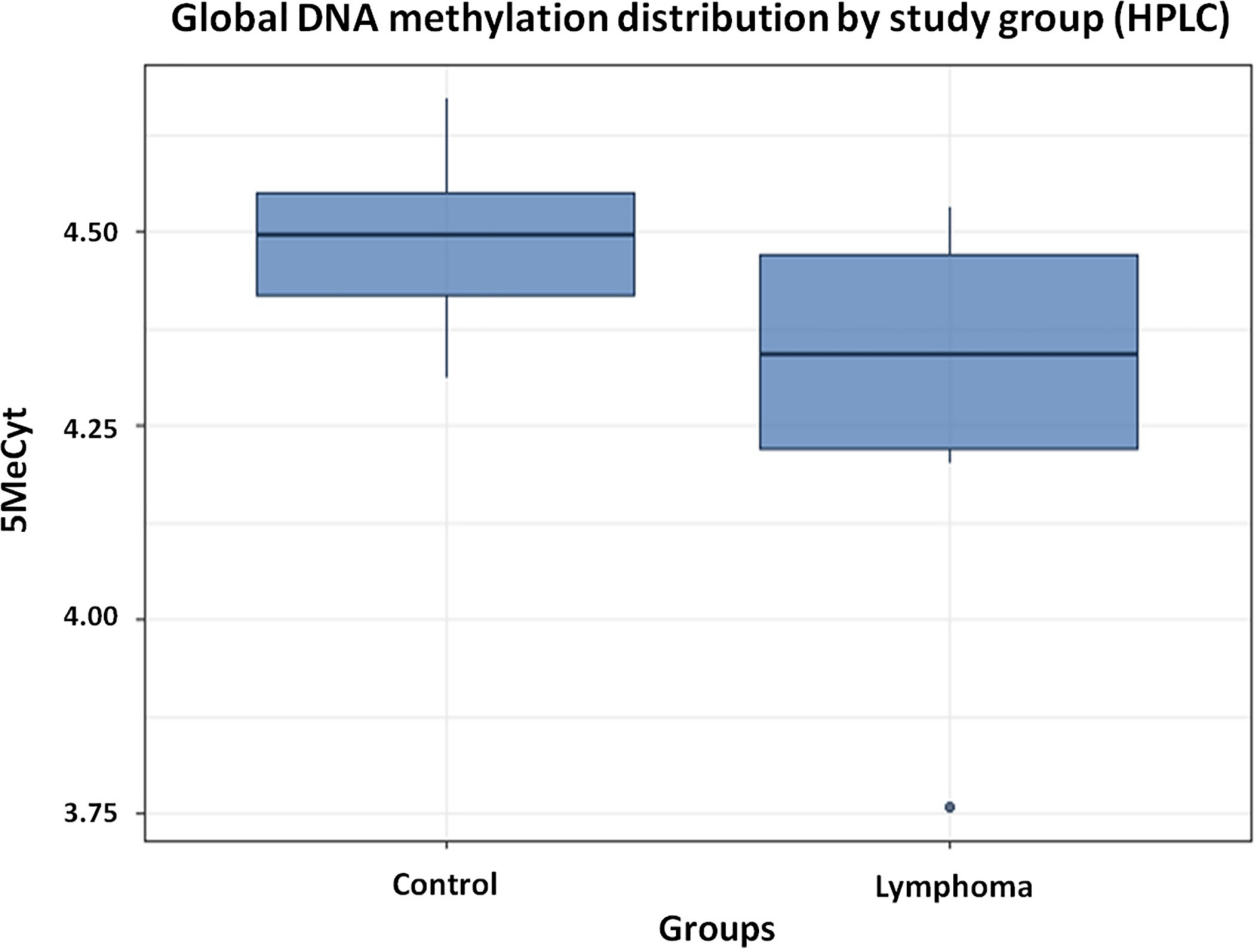
Association between levels of DNA methylation (% 5MeCyt/5MeCyt+dC) for experimental group (n = 9) compared with control group (n = 10). Student’s *t*-test (*p*-value<0.05/95% CI).

Analysis of the chromatograms showed that dogs with lymphoma (experimental group) presented reduced global DNA methylation compared with that of healthy dogs (control group) (4.29 ±0.24 vs. 4.49 ±0.10, expressed as % 5MeCyt/ 5MeCyt+dC, *p*-value = 0.027) (Table 2, S1 Table and Fig 2).

**Table 2:** Control and experimental groups for global DNA methylation quantification by HPLC and immunoreactivity for 5MeCyt.

	HPLC (% 5MeCyt/ 5MeCyt+dC)		Immunoreactivity (5-methylcytosine)	
	Control	Lymphoma	Control	Lymphoma
	(n = 10)	(n = 9)	(n = 10)	(n = 9)
Average	4.49	4.29	227.5	208.7
Median	4.49	4.34	226.5	213.8
Minimum	4.31	3.76	196.6	181.4
Maximum	4.67	4.53	253.6	224.3
Standard deviation	0.1	0.24	15.9	14.1

Two-way ANOVA test reinforced those results and demonstrated that global DNA methylation was statistically lower in lymphoma than control groups (*p*-value = 0,0298) even when those effects were evaluated simultaneously with sex, which was not statistically significant (*p*-value = 0,4243); the same was observed when age were evaluated (*p*-value = 0,6011) together with global DNA methylation by HPLC (*p*-value = 0,0315).

Moreover, there was no statistical difference in the lymphocytes counts circulating between the lymphoma and control groups by the Mann-Whitney test (*p*-value = 0.8364). In addition, no correlation was observed between the global methylation (HPLC) and the lymphocytes count by Spearman test (correlation coefficient: -0,01; *p*-value = 0.9573).

#### ICC (5-methylcytosine)

**Fig 3:**
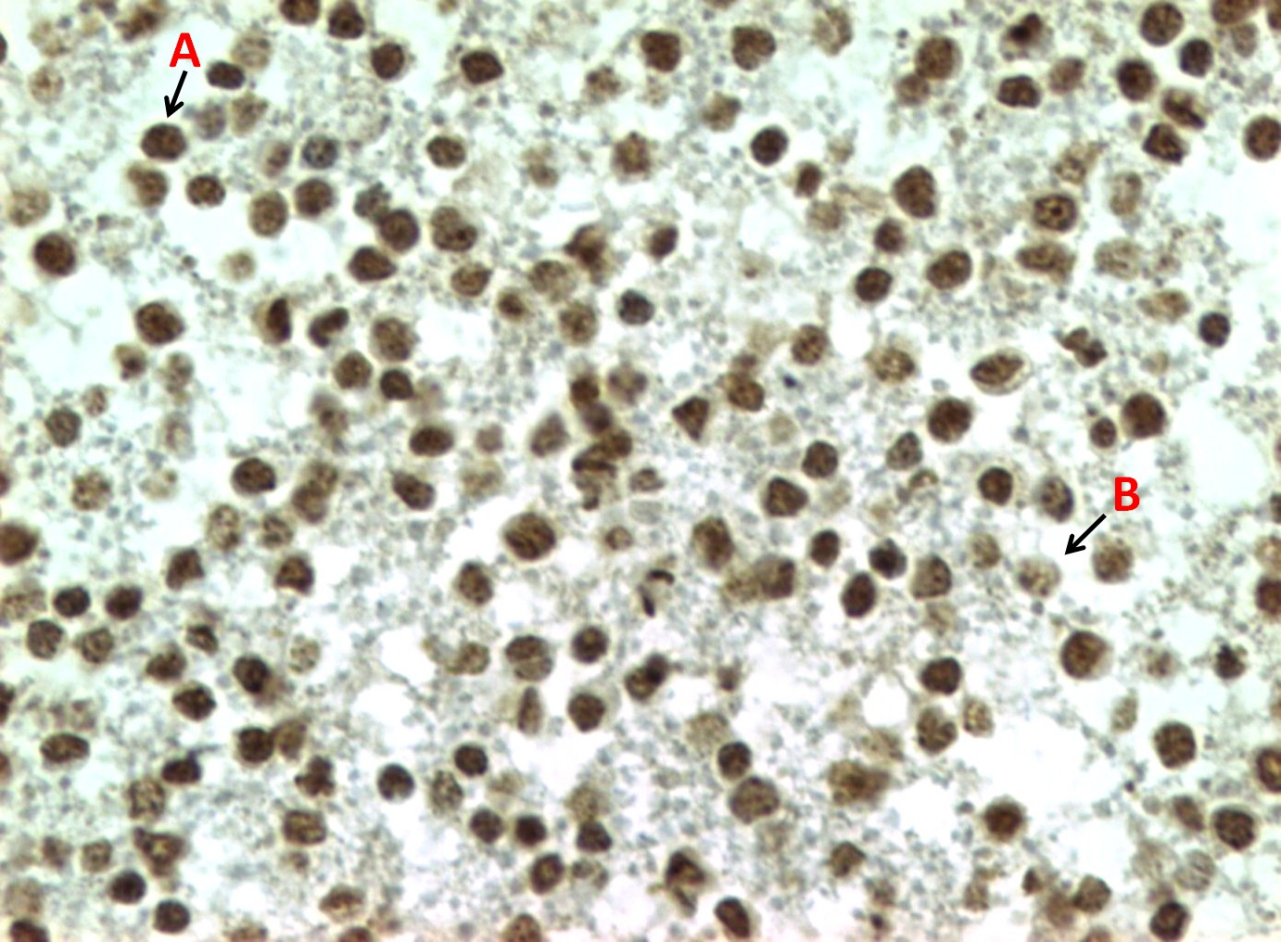
Representative example of ICC for 5-methylcytosine in a CB produced by leukocytes from a dog with lymphoma. Different grades of staining intensity caused by anti-5-methylcytosine antibody. A: strong immunoreactivity and B: weak immunoreactivity (40x objective).

Immunoreactivity for 5-methylcytosine is localized in the nuclei of the cells, and they were distinctly different between leukocytes from the same sample, characterized by nuclei labeled darker and uniformly stained (strong immunoreactivity) and dotted nuclei (weak immunoreactivity) (Fig 3).

**Fig 4:**
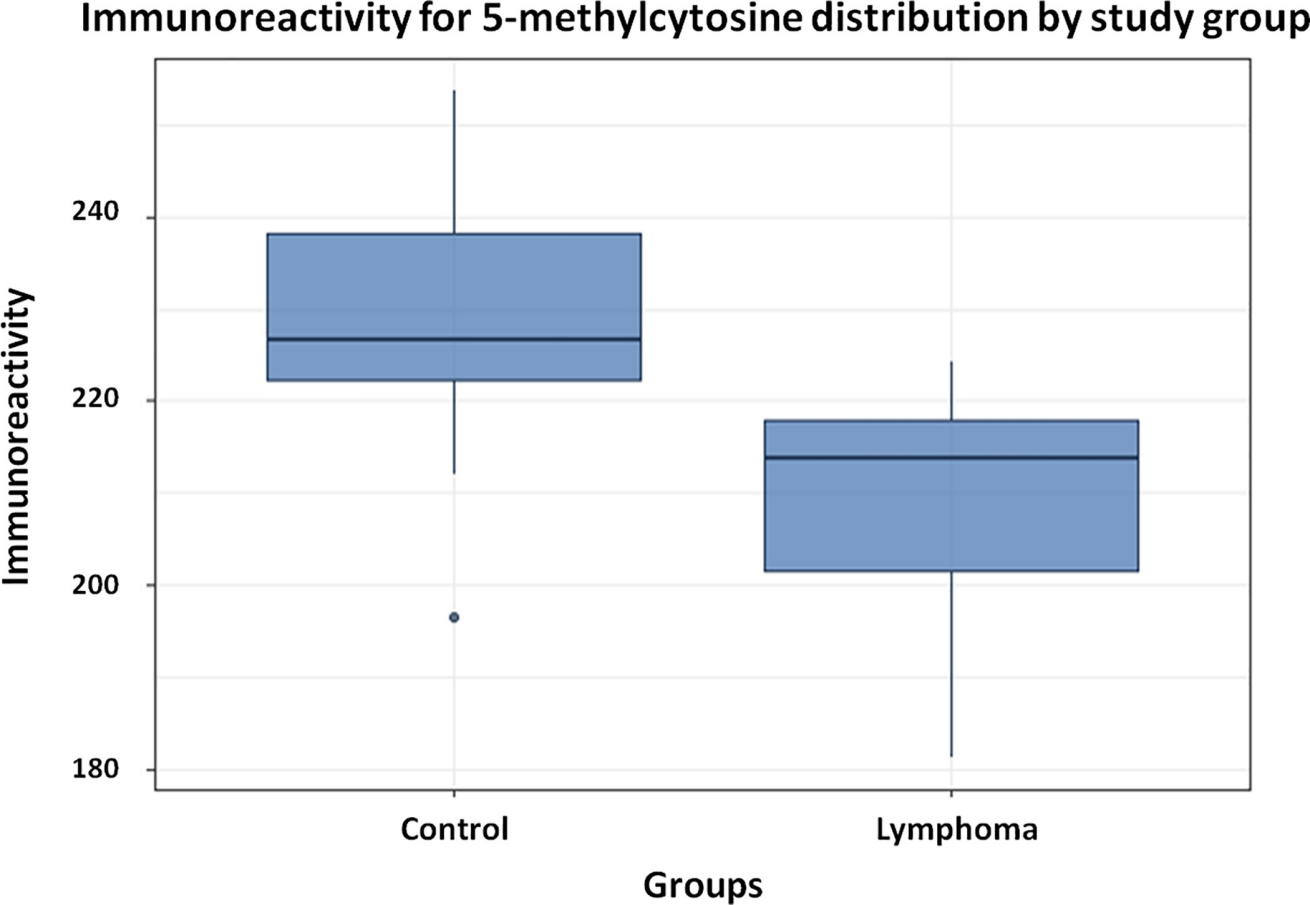
Graphic representation of immunoreactivity for 5-methylcytosine in leukocytes of the experimental group (n = 9) compared with that in the control group (n = 10). Student’s *t*-test (*p*-value<0.05/95% CI).

Immunoreactivity for 5-methylcytosine in leukocytes with weak immunostaining pattern were found in significantly higher numbers in the lymphoma group compared with that in the control group (208.7 ±14,1 vs. 227.5 ±15.9, *p*-value = 0.015) by the Student’s t-test (Table 2, S1 Table and Fig 4).

Two-way ANOVA test reinforced those results and demonstrated that global DNA methylation was statistically higher in lymphoma than control groups (p-value = 0,0181) even when those effects were evaluated simultaneously with sex which was not statistically significant (p-value = 0,7525); the same was observed when age were evaluated (p-value = 0,4583) together with global methylation (p-value = 0,0168). Moreover, no correlation was observed between the global DNA methylation (ICC) and the number of circulating lymphocytes by Spearman's correlation (correlation coefficient: -0.147; p = 0.545).

#### Correlation between global leukocyte DNA methylation quantification methods (HPLC and ICC)

**Fig 5:**
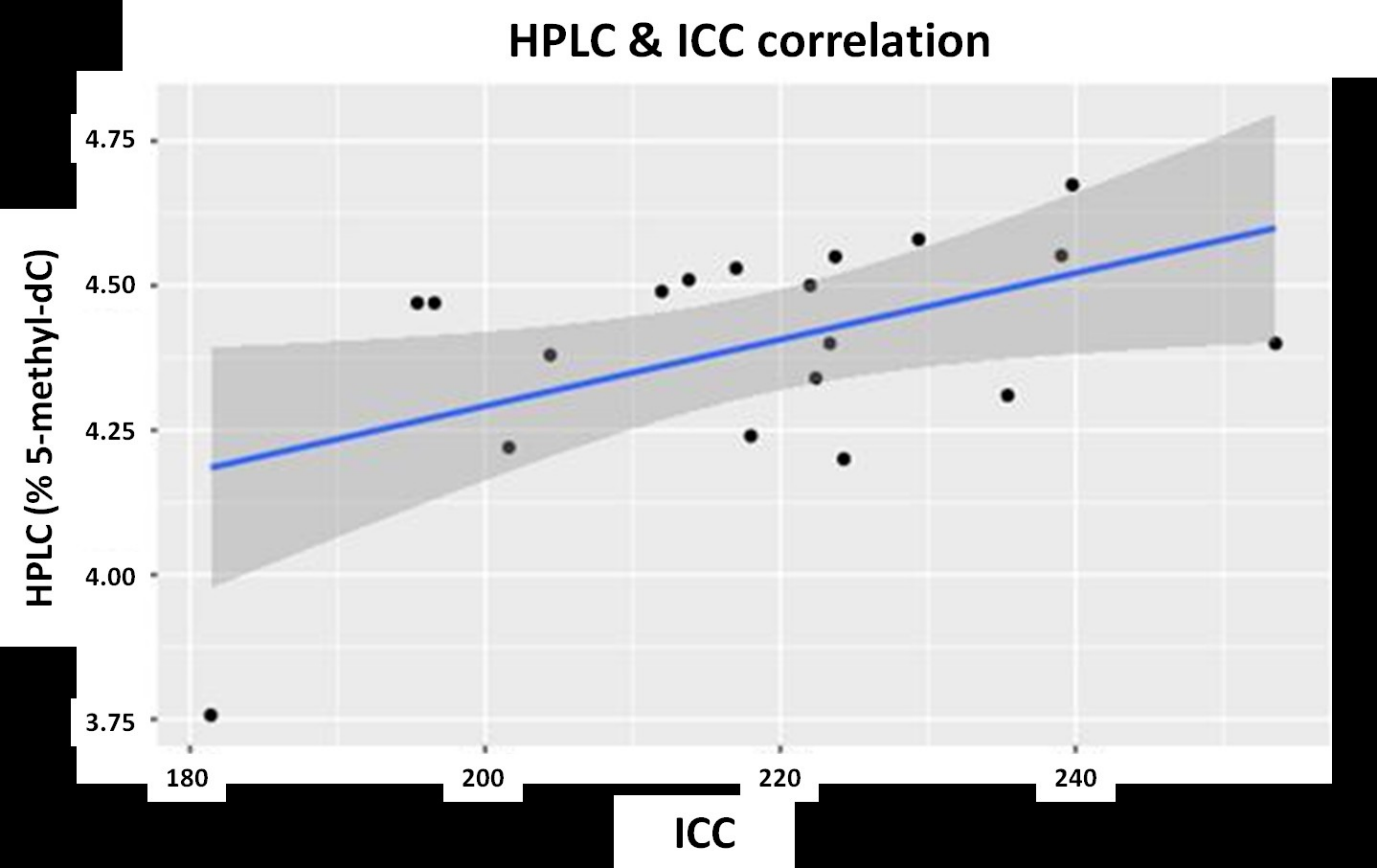
Graphic representation of the Pearson correlation between the HPLC and ICC global leukocyte DNA methylation quantification methods. *p*-value<0.05/95% CI.

Moderate positive correlation was observed between both quantification methods of global leukocyte DNA methylation. The Pearson correlation is 0.50 (*p*-value = 0.029) (Fig 5).

### STAGE 2: Infinium HM850 BeadChip DNA methylationEPIC analysis

#### Sampling

Four dogs with mean age of 9.25 years (5–11 years), one male and three females, belonged to the Control group. Dog breeds were Golden Retriever (100%). Four dogs bearing high-grade B-cell lymphoma, with mean age of 10.5 years (6–13 years), three males (75%) and one female (25%), were included in the Experimental group, composed of one mongrel and dogs of the following breeds: Labrador (1), Golden Retriever (1), and Maltese (1) (Table 3 and S1 Table).

**Table 3:** Demographic characteristics and lymphocytes counts circulating of Stage II participants (*n* = 8).

	ID	Breed	Gender	Age (years)	Lymphocytes (/μL)*	Lymphocytes (morphology)
Lymphoma	L-01	Labrador	F	11	808	Normal cell morphology
	L-03	Maltese	M	12	4,580	Rare reactive lymphocytes
	L-05	Golden Retriever	F	6	1,350	Normal cell morphology
	L-08	Mongrel	F	13	944	Normal cell morphology
**Average ± SD**				**10,5 ± 3,1**	**1.921 ± 1.787,9**	
Healthy Controls	C-04	Golden Retriever	F	11	2,208	Normal cell morphology
	C-07	Golden Retriever	F	11	1,863	Normal cell morphology
	C-08	Golden Retriever	F	5	3,212	Normal cell morphology
	C-10	Golden Retriever	M	10	2,324	Normal cell morphology
**Average ± SD**				**9,25 ± 2,8**	**2.402 ± 574,5**	

M = male, F = female

*Reference interval [48]: 1.000–4.000/ μL

#### Illumina 850K methylation

**Fig 6:**
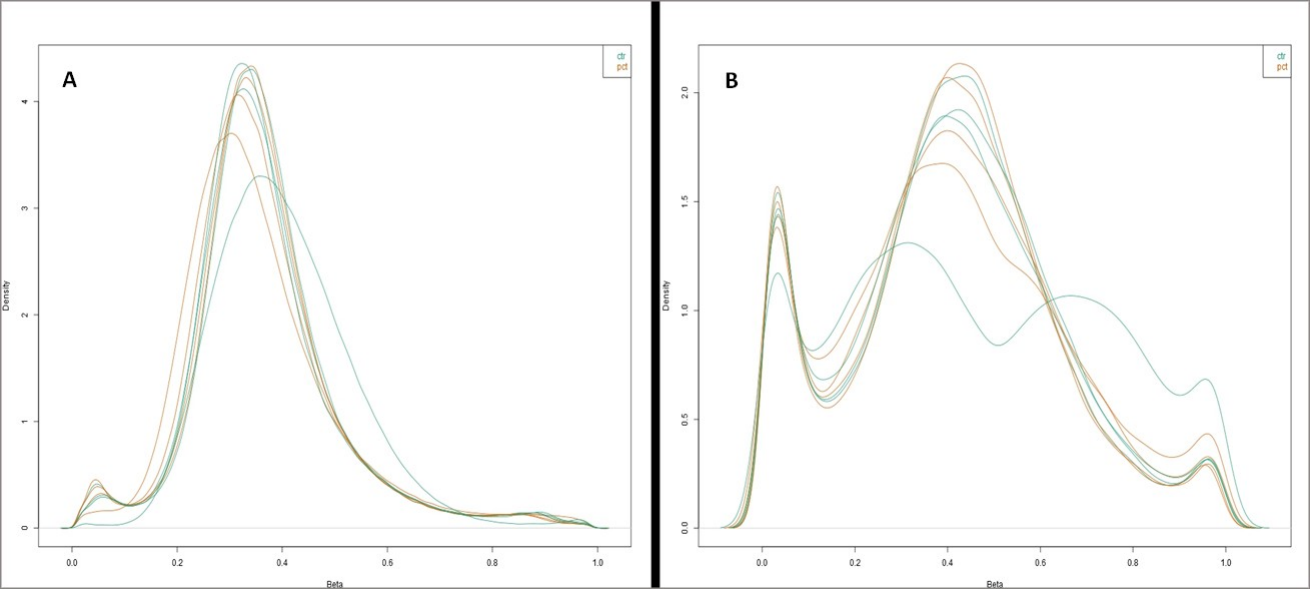
Beta score distribution of the 34,574 probes found on the HM850 BeadChip DNA methylation. The shapes of the distribution curves from all the samples of the control group were comparable to those of the experimental group. A: samples before normalization and B: samples after normalization. Ctr: samples of the control group; Pct: samples of the experimental group.

Probes that failed during the hybridization phase were removed. As a first unbiased approach, the detection signal of all EPIC probes was examined and a total of 276.592 were identified. After the normalization processes, the number of CpG sites was reduced, and only the 34,574 probes that were presented in the eight canine samples were considered (S2 Table). After that, the performance of these probes was assessed by plotting the distribution of Beta scores for 34.574 probes, which revealed a distinct distribution curve for the samples analyzed when the control and experimental groups were compared. With the distribution peaking at a Beta score of 0.3, such a curve in human sample analysis is indicative of failed analysis [49]. However, plotting the beta distribution of the subset of 34,574 probes, the distribution curves from the two group samples formed an acceptable shape according to quality control (Fig 6).

Of the 34,574 probes that have been taken to measure DNA methylation in dog samples, the standard deviation of Beta scores was calculated for each probe across the control and experimental groups. According to Needhamsen and colleagues, there is limitation to the use of EPIC for certain applications such as detection of differentially methylated regions (DMRs) because each gene is targeted by only a few probes in mouse, which was also found in samples of dogs [41]. Comparative analysis revealed 8433 regions with high variation of Beta score across the groups, corresponding to 748 genes (*p*-value<0.05) (S3 and S4 Tables). Next, as the HM850 is not specific for canine DNA and the probes are driven to the human genome (*hg19*), homology inference of the genes was performed by UCSC Genome Browser and orthology inference was performed by Ensembl database. Of the 748 genes, 525 showed homologous and orthologous similarities, including 89 genes differently methylated that exhibited *p*-value<0.01 between the study groups (S5 Table). Some highlighted genes are displayed in Table 4, presenting hypermethylated or hypomethylated cases of lymphoma compared with the control.

**Table 4:** List of hypermethylated and hypomethylated probes in the experimental group in comparison with the control group (*p*<0.05).

ID Probe	Gene	Gene description	logFC	P-value
cg26144202	*KCTD11*	potassium channel tetramerization domain containing 11	-564,935,117,912,232	0.0001
cg07584494	*KDM4B*	lysine demethylase 4B	-40,214,998,817,122	0.0006
cg05926784	*RFX1*	regulatory factor X1	-232,865,135,236,799	0.0008
cg00913953	*CHD5*	chromodomain helicase DNA binding protein 5	-219,793,357,460,489	0.0011
cg19329121	*PDK4*	pyruvate dehydrogenase kinase 4	-191,159,241,211,223	0.0025
cg09073539	*PAX2*	paired box 2	-19,110,476,323,946	0.0036
cg04205107	*PLD5*	phospholipase D family member 5	-169,503,946,066,706	0.0047
cg17804348	*TP73*	tumor protein p73	-15,990,516,386,811	0.0054
cg11980500	*TBC1D16*	TBC1 domain family member 16	-189,968,102,538,758	0.0056
cg01083397	*MECP2*	methyl-CpG binding protein 2	-176,306,255,238,775	0.0065
cg20674490	*RUNX3*	runt related transcription factor 3	-189,249,421,576,613	0.0106
cg04709321	*RASGRF2*	Ras protein specific guanine nucleotide releasing factor 2	-144,343,103,706,994	0.0218
cg17983217	*DDAH2*	dimethylarginine dimethylaminohydrolase 2	-134,883,058,962,272	0.0228
cg05886671	*NEUROG3*	neurogenin 3	-121,890,262,341,614	0.0231
cg08101036	*HOXA3*	homeobox A3	-132,568,065,256,385	0.0251
cg06829968	*ONECUT1*	one cut homeobox 1	-110,506,200,031,854	0.0260
cg01003015	*VIM*	vimentin	-111,695,841,212,767	0.0336
cg09621572	*LTA*	lymphotoxin alpha	-110,876,080,768,704	0.0410
cg16788538	*BCL7B*	BCL tumor suppressor 7B	-107,969,136,790,801	0.0451
cg22281380	*GRB10*	growth factor receptor bound protein 10	173,156,804,546,141	0.0034
cg09075558	*WNT3A*	Wnt family member 3A	179,660,497,849,853	0.0243
cg25504403	*ORAOV1*	oral cancer overexpressed 1	111,866,817,823,371	0.0267
cg26973266	*TRAF4*	TNF receptor associated factor 4	153,777,903,024,194	0.0298
cg17337672	*FGFR2*	fibroblast growth factor receptor 2	151,069,810,097,825	0.0341
cg06382559	*TLX1*	T-cell leukemia homeobox 1	121,422,502,873,281	0.0413
cg09679690	*CDK5*	cyclin dependent kinase 5	111,973,892,685,677	0.0420

ID probe—Differently methylated site; Gene: probe-associated gene; logFC: *M-value* demonstrating the difference of methylation between the control and experimental groups, positive values indicate hypermethylated probes and negative values indicate hypomethylated probes in the control group compared with those in the experimental group.

**Fig 7:**
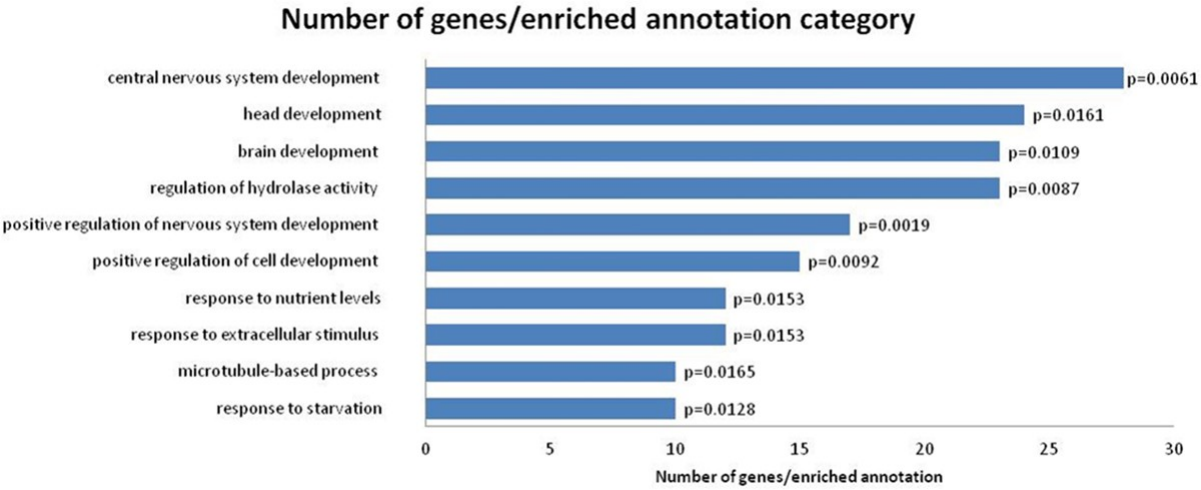
Gene ontology enrichment for hypermethylated genes.

For hypermethylated genes of the experimental group, functional annotation enrichment analysis was performed and the significant annotation categories are presented in Fig 7. Enriched annotations were related to Biological Process: developmental processes, regulation of cell development and response to external stimulus.

## Discussion

Results of this case-control study demonstrated that global methylation is detectable in DNA extracted from canine peripheral blood samples. In addition, lymphoma group leukocytes showed global DNA hypomethylation compared with healthy group leukocytes, suggesting that dogs with lymphoma present a systemic decrease in overall DNA methylation. The terms hypo- and hypermethylation refer to "less" or "more" methylation compared to standard DNA. In the case of epigenetic cancer, the pattern is established by DNA from healthy cells or tissues [50].

HPLC and immunocytochemistry techniques were effective to quantify 5MeCyt in canine peripheral blood leukocytes. Although HPLC required a large amount of extracted DNA, DNA extraction and its processing have been efficient in canine leukocytes, as well as the sample volume and storage time were adequate and able to be reproduced and used routinely. The use of a low-cost technique, initially demonstrated in neoplastic cells, provided efficient inclusion of leukocytes in paraffin and the nuclear immunostaining of the anti-5-methylcytosine antibody enabled visualization of immunostaining intensity score pattern associated with preserved cellular integrity [39]. The use of immunoreactivity scores enabled observation of dark and uniform or punctual markers delimited by weak labeling in the leukocyte nuclei, suggestive of hypermethylation of chromatin and hypomethylation, respectively, characteristics described in neoplastic tissues [51]. Hernandez-Blazquez and colleagues (2000) and Morimoto and colleagues (2016) showed that the most aggressive tumors present lower levels of global DNA methylation in immunohistochemical analysis of neoplastic tissues. The present study suggests that this aberrant methylation can also be observed in circulating leukocytes. Moderate correlation was observed between the results obtained by the ICC and HPLC (“golden method") in the quantification of global methylation methods. Therefore, the ICC method visually reflected the results obtained by HPLC. In medicine, advances in epigenetic technologies (often utilizing array-based methylation assays) have offered the possibility of examining the epigenetic state at many different *loci* associated with a cancer phenotype simultaneously in a large number of individuals [51,32].

The results of this study are in accordance with those of the study by Frizo and colleagues (2013), presenting global hypomethylation in leukocytes of humans with different types of cancer (hematological, urinary, gastrointestinal, prostate, breast, kidney, lung, and larynx) by the liquid chromatography/mass spectrometry (LC/MS) method [52]. Another recent study showed that global DNA hypomethylation, detectable in peripheral blood, is an early event in human MBCN development [22].

DNA methylation has been intensively investigated as a potential epigenetic biomarker for cancer disease and carcinogenesis; however, the true rule of global hypomethylation during cancer development is still questionable. Studies reinforce the idea that there is a common epigenetic basis during the pathogenesis and progression of distinct types of cancer. Animal experiments support that systemically low genomic methylation in tumor tissues may be causally involved in tumorogenesis, possibly by promoting chromosomal instability and activation of proto-oncogenes. Moreover, hypomethylation patterns may influence the mobilization of retrotransposons in human genomes [19,53,54]. Furthermore, low genomic methylation in circulating leukocyte DNA was associated with early colorectal tumorigenesis and aberrant DNA methylation seems to be an important factor in the pathogenesis of MBCN [28,55]. For these reasons, many epidemiological analyses in humans have suggested a systemic effect of peripheral blood DNA hypomethylation on predisposition to cancer, and have used leukocyte methylation as a risk marker [55,56]. In contrast, global DNA hypomethylation may also be considered a consequence of tumor, principally due to poor diet and nutrition (deficiency of the methyl group) in oncology patients [57,58]. Finally, evidence in human ovary epithelial tumors showed that DNA hypomethylation and hypermethylation coexist in the same tumor, but in different sequences, suggesting that methylation is a dynamic process [59].

In the present study, it was not possible to strengthen the role of hypomethylation as cause or consequence, or to establish a relationship between global hypomethylation in leukocytes and cancer risk. Thus, further epidemiological investigations are needed to clarify how these biological processes operate in canine leukocytes and whether global hypomethylation can affect the genomic stability and carcinogenesis in canine lymphoma and other diseases.

Interpretation of global methylation changes in peripheral blood still presents some issues. It has been suggested that immunologic processes associated with inflammation in cancer development lead to changes in leukocyte subpopulations, which could alter the epigenetic signatures in DNA from peripheral blood. Therefore, aberrant methylation could reflect co-incident events in the development of lymphoma, such as inflammatory or immune response [60,22]. In the present study, although the average of circulating lymphocytes count from dogs bearing lymphoma group was higher than healthy group, there was no statistical difference between the two groups and no correlation was observed between amount of global methylation and number of circulating lymphocytes. The identification of specific regions with DNA methylation changes in dogs with cancer and inflammatory diseases is also required to better understand the mechanisms of these diseases and has potential to provide novel markers for diagnosis and prognosis.

Age is a relevant factor in the methylation pattern, and human genome-wide studies have revealed a decrease in general methylation with aging [61]. Zheng and contributors (2016) concluded that age-related epigenetic blood changes may reflect cancer-related epigenetic abnormalities, serving as a biomarker for early detection of cancer [62]. In particular, hematopoietic stem cell declines in aging individuals were described as determined by age-dependent changes in DNA methylation. Ferraresso and colleagues (2014) described the first indication of age-associated epigenetic modifications in tumor tissue from canine DLBCL [37]. Ito and collaborators measured methylation in seven age-related DNA regions in canine peripheral blood leukocytes, significant correlations between methylation level and age were identified in four regions in the samples obtained from diseased dogs; however, correlations were detected only in two regions in the samples from healthy dogs [63]. Poor health status, especially in the case of age-related diseases, may cause methylation patterns that mimic age-related changes in methylation [63]. A study in humans found that some age-related methylation changes become insignificant after restricting the study sample to those without history of major age-related diseases, such as diabetes mellitus, cardiovascular disease, stroke and cancer [64]. In this research, the mean age of dogs with lymphoma (10,5 years) was greater than that of dogs in the control group (7.7 years), but the global DNA methylation was not statistically significant when ages were evaluated. Indeed, these outcomes corroborate with the studies above and suggest that global hypomethylation observed in dogs bearing lymphoma seems not to be related to the age.

HPLC results motivated an additional design in this analysis. In human medicine, advances in epigenetic technologies based on hybridization in microarrays enable search of the epigenetic state at different *loci* associated with the cancer phenotype. This technique is directed to two types of data: gene clustering (discovery of methylation profiles) and identification of unusual patterns in the selection of genes of interest [65]. When this project started, there was no specific canine microarray test commercially available and Wong and colleagues (2013) demonstrated that the Infinium Human Methylation BeadChip arrays had utility for methylation profiling in non-human species [49]. Therefore, based on research mentioned, for the comparative analysis of DNA methylation profiles of leukocytes from dogs bearing lymphoma and healthy dogs, the Infinium Human Methylation 850 BeadChip EPIC analysis was used. In this research of the 853,307 CpG islands, there was hybridization of 34,574 probes in all canine samples, in accordance with Needhamsen and contributors (2017), who described 33,100 probes with uniquely mapped hits by mappability of HM850 EPIC probes for the dog genome [41]. Additionally, Needhamsen et al. (2017) investigated the usefulness of the Infinium HM850 BeadChip EPIC platform to examine DNA methylation in mouse samples, and showed that a subset of probes found in this matrix (19,420 probes) was capable of demonstrating DNA methylation profiles in the mouse genome, being a viable and accessible option for this species and probably for another laboratory species such as Rat, Guinea pig, Rabbit, Sheep, Pig, Cow, Dog, Cat, Macaque, and Chimpanzee [41].

Results of this study demonstrated that leukocytes from dogs bearing lymphoma are characterized by widespread aberrant methylation affecting 8433 regions, corresponding to 525 genes (*p*-value<0.05) with homologous and orthological similarities (343 hypermethylated and 182 hypomethylated genes in experimental vs. healthy groups). In a comparative study between human and canine homologous genes, 5331 genes (52.3%) presented CpGs islands associated with the promoter region present in both genomes [66]. Because of their localization, these islands may be associated with gene expression, and hypermethylation of these sites is correlated with changes in transcriptional regulation, either for gene inactivation (promoter regions) or for stimulated transcription (gene body) [67]. Hypomethylation in specific promoter regions can activate aberrant expression of oncogenes and induce imprinting loss in some *loci* [67].

In addition, the GO enrichment analysis was performed to reveal the function of proteins encoded by genes showing DNA methylation alterations shared by dogs bearing lymphoma and to reveal the biological processes in which such proteins participate. This analysis demonstrated that DNA hypermethylation was enriched among development processes, regulation of cell development and response to external stimulus. Of the 525 genes related to the differently methylated sites, three genes that were hypermethylated in the experimental group compared with the healthy group (*p*-value<0.001) are worth highlighting: *KCTD11* is a tumor suppressor gene, some types of cancer present its expression decreased, such as prostatic adenocarcinoma and human medulloblastoma [68]; *RFX1* is a transcription factor, often epigenetically silenced in human glioblastomas, it directly regulates the expression of CD44, this mechanism may contribute to the proliferation and invasion of glioblastoma cells [69]; *KDM4B* is a protein involved in histone demethylation and plays a role in the carcinogenesis of many solid tumors, its expression may be associated with an aggressive subtype of non-Hodgkin lymphoma and involved with radio resistance [70].

In the analysis of DNA methylation patterns between the experimental and control groups, some genes related to the progression and development of cancer (*p*-value<0.05) were hypermethylated in dogs with lymphoma compared with the healthy group: *RUNX3* is a tumor suppressor gene, member of the *RUNT* transcription factor family, in human gastric cancer, it is often inactivated by loss of alleles or by gene silencing secondary to promoter region hypermethylation [71]; *RASGRF2* is a tumor suppressor gene that plays a key role in lymphocyte proliferation, T cell signaling, and lymphomagenesis [72]. Hypermethylation of *RASGRF2* has been associated with recurrence of prostatic cancer [73]; *BCL7B* is a member of the *BCL7* gene family (along with the genes *BCL7A* and *BCL7C*), in humans, the decrease in *BCL7A* expression is associated with development of NHL and epithelial lymphoma [74,75]. Deletion of *BCL7B* in patients with Williams-Beuren syndrome increases the risk of malignant transformation. *BCL7B* negatively regulates the WNT signaling pathway (associated with cell proliferation), and positively regulates the apoptotic pathway [76].

Hypomethylated genes in the experimental vs. healthy groups included: *TRAF4*, member of the *TRAF* family, plays an important role in the regulation of survival, cell proliferation, and stress response. Hyperexpression of *TRAF4* has been described in human marginal zone lymphomas, highlighting its role as transcription factor in lymphoma pathogenesis [77]. Reduction of *TRAF4* expression promoted inhibitory effects on the proliferative ability of osteosarcoma cell culture [78]; *CDK5*, member of the family of cyclin-dependent kinases, plays a role in DNA damage response and cell cycle checkpoint activation. *CDK5* hypomethylation has been described in mantle cell lymphoma [79]. Patients with diffuse large B-cell lymphoma present a greater expression of *CDK5* compared with that of healthy individuals [80].

In a recent research, Ferraresso and colleagues (2017) investigated the DNA methylome in tumor tissues primary from canine DLBCLs in comparison with control lymph nodes using for the first time a DNA CpG microarray designed for canine species [31]. The accumulation of aberrant epigenetic changes in the tumor samples resulted in a more aggressive behavior of the subtypes of canine diffuse large B-cell lymphomas, and gene functional analysis highlighted biological processes strongly associated with embryonic development, tissue morphogenesis, and cellular differentiation, including *HOX*, *BMP* and *WNT* [31]. Interestingly, compared with our study, although different techniques and samples types (tumors and circulating leukocytes) were used, some genes described by Ferraresso and colleagues in tumor also presented aberrant methylation in canine leukocyte DNA (KDM4B, HOX, WNT and FGFR2) [31]. According to two studies about utility of human DNA methylation arrays for profiling genomic DNA in other species seems that human methylation chip could be an option for canine methylome studies in the commercial unavailability of the canine chip [41,49].

As several differently methylated genes have not yet been described in canine high-grade B-cell lymphoma, it is important to validate our findings in larger studies conducted to detect differently methylated regions and specific *loci* of each of the 525 genes described, by PCR and sequencing techniques, in addition to verify their gene expression in neoplastic tissues and cell cultures. This follow-up may identify aberrant methylation in specific regions of functional importance and improve understanding of the role of DNA methylation in lymphomas, as well as the search for new possible biomarkers for canine lymphomas.

Although a larger number of samples both from healthy dogs and dogs bearing lymphoma are needed, this study emphasizes the importance of researching methylation in leukocytes, mainly by easily accessing samples via non-invasive collection. In addition, our results reiterate the importance of the use of liquid biopsy in cancer research; the use of peripheral blood instead of tumor tissue presents as main advantages the easy access and the possibility of collecting samples from the same patient at different times, making it possible to use them as diagnostic biomarkers and to monitor them during treatment.

Finally, this pioneer study associated canine lymphoma with global hypomethylation of DNA and aberrant methylation patterns in leukocytes obtained from peripheral blood. Epigenetic similarities between the human and canine species increase the relevance, in the field of cancer research, of the identification of epigenetic modifications, cancer-associated genes, the study of environmental risk factors and, most importantly, the evaluation and development of novel cancer biomarkers of canine non-Hodgkin lymphoma.

## Supporting information

S1 Table Complete clinical and pathological features of dogs included in study. (DOCX)

S2 Table Probes that were presented in all canine samples (n = 8). (XLSX)

S3 Table Regions with high variation of Beta score across the groups. (XLSX)

S4 Table Genes contained in regions with high variation of Beta score across the groups (*p*-value<0.05). (XLSX)

S5 Table Genes that showed homologous and orthologous similarities (*p*-value<0.05). (XLSX)
