# Supplementary material for: Global DNA methylation of peripheral blood leukocytes from dogs bearing multicentric non-Hodgkin lymphomas and healthy dogs: A comparative study
Source: PLoS One. 2019 Mar 25;14(3):e0211898. doi: 10.1371/journal.pone.0211898 (PMC6433272; doi:10.1371/journal.pone.0211898)
Supplement: Supplementary file 1 [file pone.0211898.s001.docx]

	ID	Breed	Gender	Age (years)	Lymphocytes (/µL)**	Lymphocytes (morphology)	HPLC (% 5-mdC)	ICC	Lymphoma (classification)	Cell type	Grade	Ki67 (%)
Lymphoma	L-01*	Labrador	F	11	808	Normal cell morphology	4,53	217,0	Centroblastic polymorphic small and large cell	B	High	67,0
	L-02	Mongrel	M	10	2.684	Normal cell morphology	4,34	222,4	Plasmocytoid	T	High	8,0
	L-03*	Maltese	M	12	4.580	Rare reactive lymphocytes	4,24	218,0	Immunoblastic	B	High	40,0
	L-04	Dachshund	F	13	26.908	Rare reactive lymphocytes	4,51	213,8	Lymphoblastic	T	High	57,0
	L-05*	Golden Retriever	F	6	1.350	Normal cell morphology	4,22	201,6	Plasmocytoid	B	High	38,0
	L-06	American Pittbul	F	13	4.440	Rare reactive lymphocytes	3,76	181,4	Centroblastic-centrocytic	B	Low	3,0
	L-07	Swiss Sheppard	F	5	891	Normal cell morphology	4,47	195,4	Centroblastic monomorphic	B	High	67,4
	L-08*	Mongrel	F	13	944	Normal cell morphology	4,38	204,4	Centroblastic monomorphic	B	High	68,0
	L-09	Mongrel	M	12	840	Normal cell morphology	4,20	224,3	Centroblastic monomorphic	B	High	23,0
Healthy controls	C-01	Golden Retriever	F	5	2.555	Normal cell morphology	4,49	212,0				
	C-02	Boxer	M	7	2.442	Normal cell morphology	4,31	235,4				
	C-03	Golden Retriever	F	5	1.380	Normal cell morphology	4,58	229,4				
	C-04*	Golden Retriever	F	11	2.208	Normal cell morphology	4,67	239,8				
	C-05	Golden Retriever	M	5	2.660	Normal cell morphology	4,50	222,0				
	C-06	Golden Retriever	F	8	930	Normal cell morphology	4,40	253,6				
	C-07*	Golden Retriever	F	11	1.863	Normal cell morphology	4,55	239,1				
	C-08*	Golden Retriever	F	5	3.212	Normal cell morphology	4,55	223,7				
	C-09	Golden Retriever	F	10	1.840	Normal cell morphology	4,40	223,4				
	C-10*	Golden Retriever	M	10	2.324	Normal cell morphology	4,47	196,6				

M=male, F= female *Dogs selected for the Stage II, **Reference interval [48]: 1.000-4.000/ µL
